# The Effect of Low-Temperature Thermal Processing on Bovine Hydroxyapatite Bone Substitutes, toward Bone Cell Interaction and Differentiation

**DOI:** 10.3390/ma15072504

**Published:** 2022-03-29

**Authors:** Gemma Claire Porter, Dina Abdelmoneim, Kai Chun Li, Warwick John Duncan, Dawn Elizabeth Coates

**Affiliations:** Sir John Walsh Research Institute, Faculty of Dentistry, University of Otago, Dunedin 9016, New Zealand; abddi084@student.otago.ac.nz (D.A.); kc.li@otago.ac.nz (K.C.L.); warwick.duncan@otago.ac.nz (W.J.D.); dawn.coates@otago.ac.nz (D.E.C.)

**Keywords:** bone graft, thermal effects, bone cells, regenerative materials

## Abstract

Ideal bone grafting scaffolds are osteoinductive, osteoconductive, and encourage osteogenesis through the remodeling processes of bone resorption, new bone formation, and successful integration or replacement; however, achieving this trifecta remains challenging. Production methods of bone grafts, such as thermal processing, can have significant effects on the degree of cell-surface interactions via wide-scale changes in the material properties. Here, we investigated the effects of small incremental changes at low thermal processing temperatures on the degree of osteoclast and osteoblast attachment, proliferation, and differentiation. Bovine bone scaffolds were prepared at 100, 130, 160, 190, and 220 °C and compared with a commercial control, Bio-Oss^®^. Osteoclast attachment and activity were significantly higher on lower temperature processed bone and were not present ≥190 °C. The highest osteoblast proliferation and differentiation were obtained from treatments at 130 and 160 °C. Similarly, qRT^2^-PCR assays highlighted osteoblasts attached to bone processed at 130 and 160 °C as demonstrating the highest osteogenic gene expression. This study demonstrated the significant effects of small-scale processing changes on bone graft materials in vitro, which may translate to a tailored approach of cellular response in vivo.

## 1. Introduction

Bone defects caused by surgery, trauma, infection, or congenital malformation often necessitate the use of bone grafting materials to act as a filler or scaffold to replace and regenerate bone [1]. In order to induce optimal bony defect repair, graft materials need a wide range of desired specifications, which include biocompatibility, resorbability, osteoconductivity, osteoinductivity, are mechanically resistant, and are easy to use, safe, and cost-effective [2,3]. With this wide range of specifications comes a range of material types and sources [4]. The gold standard, adhering to the aforementioned specifications, is autologous bone, bone harvested from the patient; however, there is the added difficulty of graft harvesting, which has been associated with pain, infection, scarring, blood loss, and donor site morbidity [5,6]. Allographs are bone materials deficient in cellular materials obtained from cadavers; however, they do not possess the osteoactivity of autographs and can carry infectious disease and cause immune rejection [7]. In addition to these, there are bone substitutes that are defined as a synthetic, inorganic, or biologically organic combination [8] and include xenogeneic bone (derived from species other than human [9]) or synthetic materials [10]. Generally, due to the ease of use, availability, and versatility, bone substitutes are a popular option. Within these bone substitutes, demineralized and deproteinized xenografts are particularly popular due to the retained natural bone structures [1]. A large proportion of available products are composed of hydroxyapatite, a naturally occurring calcium phosphate that comprises 60–70% of the mineral content of bone [11]. Hydroxyapatite can be obtained from bovine bone and is used in many commercial products (Bio-Oss^®^ (Geistlich Biomaterials, Geistlich, Switzerland), Osteograf-N™ (CeraMed Co., Denver, CO, USA), and Endobon^®^ (Merck Co., Darmstadt, Germany) [12], but also can be derived from coral, such as Interpore^®^ and Pro-osteon^®^ (Interpore International, Inc., Irvine, CA, USA), porcine, equine, or synthetic sources [11].

Subtle differences in graft production can have significant effects on the stimulatory effects of bone growth and, therefore, successful integration of the grafting material. Of these methods, the thermal treatments used to remove organic materials is a process that greatly varies the macro, micro, and nano-scale of the material and thus alters the attachment, proliferation, and differentiation of cells [13]. Typically most commercially available artificial bone substitutes are high-temperature apatites (600–1400 °C) due to the utilization of high-temperature removal of organic materials, prions, and reduction in the risk of infection [9]. The high thermal processing provides the material with increased crystallinity, with a significant change to apatite crystals occurring between 600 and 800 °C [9,14]. Higher crystallinity was associated with resistance to biodegradation, lack of degradation by osteoclasts, and limited osteoconductive activity; however, it also produces an increase in mechanical strength [15]. High-temperature treatment can also induce increased osteoconductivity through increased porosity [15,16], with pores of ≤300 µm in diameter known to promote osteogenesis [17]. However, there is no agreed optimal porosity with the literature reporting a wide range of sizes and percentage volumes [18]. The use of lower temperature processing was shown to result in apatite with more similarity to its biological form, increased surface area, and higher reactivity with cells in bone substitutes [19,20,21].

In the following investigation, prion-free New Zealand bovine bone specimens were processed using low-temperature intervals between 100 and 220 °C and were assessed for their degree of osteoclast and osteoblast attachment and differentiation. A comparison was made to a bovine bone substitute Geistlich Bio-Oss^®^, a commonly used xenograft bone material (processed between 300 °C and 500 °C [22,23,24,25,26]). In a previous in vivo ovine study of sinus bone grafting performed by the authors, Bio-Oss^®^ underwent minimal resorption by osteoclasts, whereas the New Zealand bovine bone material was rapidly resorbed; therefore, this study serves to increase the retention of the New Zealand bone material through thermal processing [27]. Ultimately, we demonstrated how small intervals at low thermal processing temperatures result in significant effects on cell activity and differentiation.

## 2. Materials and Methods

### 2.1. Bone Preparation

New Zealand bone blocks were derived from the cancellous region of bovine femurs. Tissue was removed from prion free New Zealand bovine bone blocks (25 × 25 × 25 mm) by boiling, rinsing with 100 °C (+/−5 °C) water and centrifugation (Molteno Ophthalmic Ltd. (batch no. 1810/SBE2) Dunedin, New Zealand). Cubes were then cut into smaller 8 × 8 × 8 mm cubes and 25 mm × 25 mm × 5 mm bone slices using a Struers Accutom-50 cutting machine (Struers, Denmark). Thermal processing was conducted in a custom-made cylindrical stainless-steel vessel with an external diameter of 120 mm, an internal diameter of 70 mm, and a height of 105 mm containing distilled water (80 mL). The bone cubes and slices were added and heated at a rate of 4–6 °C/min and held for 2 h at either 100 °C, 130 °C, 160 °C, 190 °C or 220 °C, producing 5 sample groups of heat-treated MoaBone (MB). Treatment was followed by a 5-minute cooling period. The pressure recorded at each temperature is presented in Table 1. Finally, the bone samples were rinsed with distilled water and air-dried for 24 h in a sterile environment at room temperature (RT). Prepared bone scaffold specimens are referred to as MB100, MB130, MB160, MB190, and MB220 for respective temperatures groups of 100 °C, 130 °C, 160 °C, 190 °C, and 220 °C processed MoaBone.

### 2.2. Cell Culture of RAW 264.7 Osteoclasts on Heated Bone

Mouse macrophage cells (RAW 264.7 (ATCC^®^ TIB-71™)) (passage 5) were grown in standard cell culture conditions (37 °C, 5% CO_2_) in cell culture medium containing DMEM (Cat. No. 10569010; Thermo Fisher, MA, USA)/10% FBS (Cat. No. F8067; Merck, NJ, USA), 50 µg/mL of gentamicin (Cat. No. 15710064; Life Technologies), and 5 mL of antibiotic– antimycotic (Cat. No. 15240062; Life Technologies Ltd., Carlsbad, CA, USA). RAW 264.7 cells are routinely used in osteoclast studies and are an important tool for in vitro studies of osteoclast formation and activation [28,29].

Bone discs were prepared from the 25 mm × 25 mm × 5 mm bone slices, using a 5.2 mm circular soft tissue punch (Ref 32Z2002 Nobel Biocare, Kloten, Switzerland), producing 5.2 mm × 5 mm discs. Prior to cell seeding, bone discs (N = 4) were sterilized by soaking in 90% EtOH (10 min, ×3), phosphate-buffered saline (PBS) washed (10 min, ×3), and a final wash performed in DMEM/10% FBS for (10 min, ×3). The bone discs were then placed onto sterile parafilm and air-dried in a sterile environment. Bio-Oss^®^ granules were also sterilized using the same methodology. Bone discs or granules were then placed into a 96 well plate in DMEM/10% FBS (100 µL) overnight at 37 °C, 5% CO_2_. After 16 hours, RAW 264.7 cells were seeded at 2000 cells per sample well (100 µL) onto the overnight-incubated bone discs containing the 100 µL of pre-incubation media to give a final volume of 200 µL. Following overnight incubation, each bone disc was aseptically moved to a 48 well plate containing αMEM (500 µL) (Cat. No. 32571036; Thermo Fisher, MA, USA) supplemented with 10% FBS, RANK-L (50 ng/mL), and colony-stimulating factor (25 ng/mL CSF). The discs were then incubated at 37 °C in 5% CO_2_ for a duration of 7 days. An additional 500 µL of supplemented αMEM 10% FBS was added to the existing media after 48 h. After 96 h, 500 µL of media was removed and replaced with fresh supplemented αMEM 10% FBS. On day 7, the specimens were analyzed for tartrate-resistant acid phosphatase (TRAP) and proliferation/morphological features via SEM analysis. A plastic well containing the same cell seeding density was used for confirmation of TRAP-positive cells.

### 2.3. TRAP Confirmation

A TRAP assay kit (Cat. No. AK04, B-Bridge International, Inc., Santa Clara, CA 95054) was utilized for staining of cells and for measurement of TRAP within the supernatant. Osteoclast differentiation was confirmed by removing the medium from the RAW 264.7 cells within the plastic well group and washing the cells with 100 µL phosphate-buffered saline (PBS) prior to TRAP staining. Cells were fixed with 10% neutral buffered formalin (50 µL) for 5 min and were washed 3 times with DIH_2_O (250 µL). Chromogenic substrate (3 mg/vial) was dissolved in Tartrate-containing buffer (5 mL) and added to the wells (50 µL) and incubated at 37 °C for 60 min. The wells were then washed with DIH_2_O. The culture media (30 µL) from each well containing RAW 264.7 bound-bone discs was transferred to a new 96 well plate. The tartrate-chromogenic substrate was added to each well (170 µL), and the reaction was left to incubate at 37 °C for 3 h. Absorbance was read using a Bio-strategy Synergy 2 Plate Reader and Gen 5 software at 540 nm.

### 2.4. SEM Analysis

Culture media was removed from the specimens used for TRAP staining, and this was replaced with 2.5% glutaraldehyde in sodium cacodylate buffer (0.19 M, pH 8.4). The plates were then placed on an orbital mixer at RT for 60 min. Cells were then washed three times for 5 min each in sodium cacodylate buffer (0.1 M) and stained using 1% osmium tetroxide (OsO_4_) in sodium cacodylate (0.1 M) for 1 h. Post staining, the cells were washed three times for 5 min each with cacodylate buffer (0.1 M). Cell bound-bone discs were then dehydrated using a graded ethanol series: 30%, 50%, 70%, 80%, 95%, and 100% for 5 min each and transferred to safe cell specimen holders, ensuring the discs and holders remained submerged in 100% ethanol. The samples were then dried using a critical point dryer with liquefied carbon dioxide as the transitional fluid. Specimens were then mounted on aluminum stubs with carbon tape and were sputter-coated with a gold-palladium mix using a Peltier-cooled high-resolution sputter coater (Emitech K575X, EM Technologies Ltd.; Kent, England). Specimens were examined using a JEOL FE-SEM 6700 (Joel Ltd.; Tokyo, Japan).

### 2.5. Cell Culture of Saos-2 Osteoblasts

The human osteosarcoma cell line (Saos-2 (ATCC^®^ HTB-85™)) (*p* = 15) was grown in standard cell culture conditions (37 °C, 5% CO_2_) in a cell culture medium containing McCoy’s (Cat. No. 36600021; ThermoFisher, MA, USA)/15% FBS, 50 µg/mL of gentamicin and 5 mL of antibiotic–antimycotic. Saos-2 cells were selected as they are known to exhibit several fundamental osteoblast characteristics and represent an accepted and representative model for in vitro osteogenic study [30,31].

Osteogenic assays were conducted on standardized bone granules (1 mm × 2 mm × 2 mm). Prior to cell assays, the granules were sterilized as described previously for bone discs. Each group of sterile bone granules (MB100, MB130, MB160, MB190, MB220, and Bio-Oss^®^, N = 4 containing 5 granules per well) were collated into one well of a 48 well plate and were pre-incubated overnight with McCoys/15% FBS. Extra granules were included in the collated groups to allow for granule loss during processing; therefore, each collated group contained ~ 30 granules. Saos-2 were seeded at 160,000 cells per well (400 µL) onto the bone particles and were incubated for 16 h. The granules were aseptically transferred to a new 48 well plate containing 500 µL of McCoys/15% FBS supplemented with 100 µM L-ascorbic acid-2-phosphatase, 10 nM dexamethasone, and 5 mM β-glycerophosphate (osteogenic media). The cell-bound granules were incubated under standard cell culture conditions over 21 days. Experimental assays were performed on day 7 and day 21.

At 7 days, a triplex assay was performed, which included confocal microscopy of NucBlue^®^ Live reagent (Hoechst 33342) and propidium iodide (PI) stained cells (ReadyProbes™ Cell Viability Imaging Kit; Thermofisher), analysis of ALP activity, and measurement of DNA content using a Picogreen DNA assay. Subsequently, at 21 days, a duplex assay was performed using confocal microscopy of DAPI and PI stained samples and analysis of DNA content using a picogreen assay. A separate set of bone particles (*n* = 5) were used for DAPI/osteocalcin immunolabelling and imaged with confocal microscopy.

At the same time points of 7 and 21 days, cells were lysed from bone granules (~30 granules per replicate group, N = 4, to allow for sufficient cells for RNA extraction) in TRIzol reagent for gene analysis (N = 4). Prior to harvesting, two of these granules were used for DAPI/Alexa Fluor™ 647 Phalloidin staining and imaging with confocal analysis.

### 2.6. Picogreen DNA Quantification

A low and high-range concentration calibration for DNA content in trypsin was performed using the picogreen assay kit, as per manufacturer instructions (P11496, Quant-iT™ PicoGreen™ dsDNA Assay Kit, Invitrogen). The bone granules were aseptically transferred from wells into separate 1.5 mL tubes and trypsin-EDTA (0.25% *v*/*v*, 200 µL) added and incubated at RT for 4 min. The bone granules were agitated to lift cells from the bone surface, and the samples were divided to allow use in the picogreen assay and the ALP assay (100 µL for each). A working solution of 1X tris-HCl EDTA (TE; 10 mM Tris-HCl, 1 mM EDTA, pH 7.5) was made, and the Quant-iT™PicoGreen^®^ reagent was diluted 200-fold in 1 X TE and stored protected from light. An equal volume of picogreen (0.1 mL) was added to the bone granule derived cell samples in a 96 well plate and incubated for 5 min at RT, protected from light. Fluorescence was measured at ex/em: 480 nm/520 nm using a Synergy 2 Plate Reader and Gen 5 software.

### 2.7. Alkaline Phosphatase Fluorometric Assay

Immediately after trypsinization of bone granule samples, 100 µL of the resulting supernatant was centrifuged at 1000× *g* for 4 min to pellet cells and bone granules. The trypsin was removed and discarded. Ice-cold PBS (200 µL) was placed onto the bone-cell pellet and then centrifuged at 1000× *g* for 4 min. PBS was removed from the bone-cell pellet and replaced with the assay buffer (100 µL), which was then pipetted up and down rapidly (ab83371 Alkaline Phosphatase Assay Kit Fluorometric, Abcam). The samples were centrifuged at 13,000× *g*, 4 °C, for 3 min, and the supernatant was collected and kept at −80 °C until required. The supernatant was allowed to equilibrate at RT prior to further analysis. Bone granules without cells that were processed through the trypsin and the centrifugation procedure were used as a background control. Assay buffer (10 µL) was added to samples (100 µL), and 4-methylumbelliferyl phosphate disodium salt (MUP; 20 µL) was added to the test/control samples and background control assay buffer. A stop solution was added to designated test background controls. Samples were incubated for 30 min at 25 °C, protected for light. The stop solution was then added to the samples, calibration standards, and background wells. The well plate was gently shaken, and fluorescence was measured using Ex/Em = 360/440 nm. A calibration of ALP enzyme concentration was conducted as directed by manufacturer instructions during the assay.

### 2.8. Live/Dead Staining of Cells on Bone Scaffolds

The culture media was removed from each well containing bone granules, and each was washed 3 times with PBS. PBS (400 µL) was placed on the specimens, and Nunc blue and PI (15 µL for each) were added to each well. The samples were incubated in the dark at RT for 30 min. Samples were subsequently washed using PBS and were maintained in 100 µL of PBS during confocal laser scanning microscopy. Image acquisition of live/dead stain, osteocalcin, and DAPI/Alexa Fluor™ assayed specimens were performed a Nikon A1+ inverted confocal laser scanning microscope (Kurobane Nikon Co., Otawara, Japan). Confocal images were analyzed for cell counts using Fiji software.

### 2.9. Osteocalcin Labelling on Cell Bound Bone Scaffolds

Immunohistochemistry was performed using osteocalcin (Cat. No. ab13421; Abcam) mAb antibody. The culture media was removed from the bone granules/Saos-2 cells after 21 days of culture, and samples were gently washed with PBS and fixed using methanol (100 µL) for 5 min. Then, they were washed again with PBS (500 µL) and incubated in tween-20 (1% in PBS) for 10 min. PBS washes prior to blocking with 20% goat serum (G9023; Sigma)/PBS (500 µL) were conducted, and the osteocalcin antibody (2.5 µg/mL in 5% goat serum/PBS (300 µL)) was applied to each sample and was left to incubate at 4 °C overnight. The samples were washed 3 times with 1% skimmed milk powder/PBS (500 µL) for 15 min each with gentle rotation. Secondary antibody (goat anti-mouse IgG secondary Dylight 488; Cat. No. NBP1-72872) (0.2 mg/mL) was incubated in the dark, and then samples were washed three times in PBS. Granules were further stained for 5 min with DAPI (300 nM, 300 µL) and washed with PBS three times.

### 2.10. DAPI/Alexa Fluor™ 647 Phalloidin

Cell bound bone granules were washed with prewarmed PBS (37 °C) and then fixed in 3.7% formaldehyde (10 min). Specimens were washed twice with PBS and ice-cold acetone placed onto the granules for 5 min. Bone granules were PBS washed and then stained for 5 min with DAPI (300 nM, 300 µL) and washed with PBS. Samples were then incubated with Alexa Fluor™ 647 Phalloidin (5 µL metholic stock solution into 1% BSA/200 µL PBS) for 20 min and were washed three times with PBS.

### 2.11. Cell Harvesting and RNA Extraction

Bone granules were sterilized as previously described and arranged as a monolayer on the base of each well of a 48 well plate (~30 granules per well) (N = 4). Each granule well was seeded with 1.6 × 10^5^ cells per well. The investigation was performed using osteogenic media and non-osteogenic media, with MB100, MB130, MB160, and Bio-Oss^®^ (N = 4 wells per group). Cells were harvested with 1 mL of TRIzol (Cat. No. 15596026; Thermo Fisher, MA, USA) per well at 7 and 21 days and were stored at −80 °C until RNA extraction was performed. Total RNA was isolated using the Invitrogen Trizol Plus RNA Purification kit and Phasemaker™ Tubes Complete system, following the recommended procedure. Genomic DNA contamination was removed using On-Column PureLink DNase treatment (Ambion, Foster City, CA, USA), and the purity and quantity of RNA were assessed using a NanoVue (GE Healthcare, Little Chalfont, UK). The RNA samples were stored at −80 °C. Total RNA (approximately 300 ng) was used to synthesize cDNA (High Capacity cDNA Reverse Transcription Kit; Gibco Invitrogen). The resulting cDNA was diluted to produce 150 μL (for high RNA-containing wells) and 40 µL (for low RNA-containing wells) quantities of 1 ng mL^−1^ using RNase-free H_2_O.

Quantitative TaqMan™ real-time PCR (qRT^2^-PCR) single-gene assays were conducted with 8 genes of interest: bone gamma-carboxyglutamate protein (*BGLAP*), integrin binding sialoprotein (*IBSP*), secreted phosphoprotein 1 (*SPP1*), collagen type I alpha 1 chain (*COL1A1*), secreted protein acidic and cysteine-rich (*SPARC*), alkaline phosphatase (*ALPL*), Sp7 transcription factor (*SP7*), X-box binding protein 1 (*XBP1*), Two housekeeping genes (HKG) of glyceraldehyde-3-phosphate dehydrogenase (*GAPDH*), and beta-2-microglobulin (*B2M*) were screened for normalization. Normfinder (Visual Basic Application applet for Microsoft Excel) was used to determine the optimal normalization gene and determined GAPDH was the most stable with an M-value of 0.057. Thermal cycling and detection were performed with a QuantStudio 6 Flex instrument (Applied Biosystems). No cDNA and no reverse transcriptase reactions were included as controls. The data were analyzed using Graphpad PRISM software (Version 6.00 for Windows, GraphPad Software, San Diego, CA, USA). Analysis of the gene assays was conducted using the raw quantification cycle (Cq) of the test genes normalized against the Cq of the reference gene using the 2^−^^△Cq^ method.

## 3. Results

### 3.1. Surface Appearance of Thermally Processed Bone

**Figure 1:**
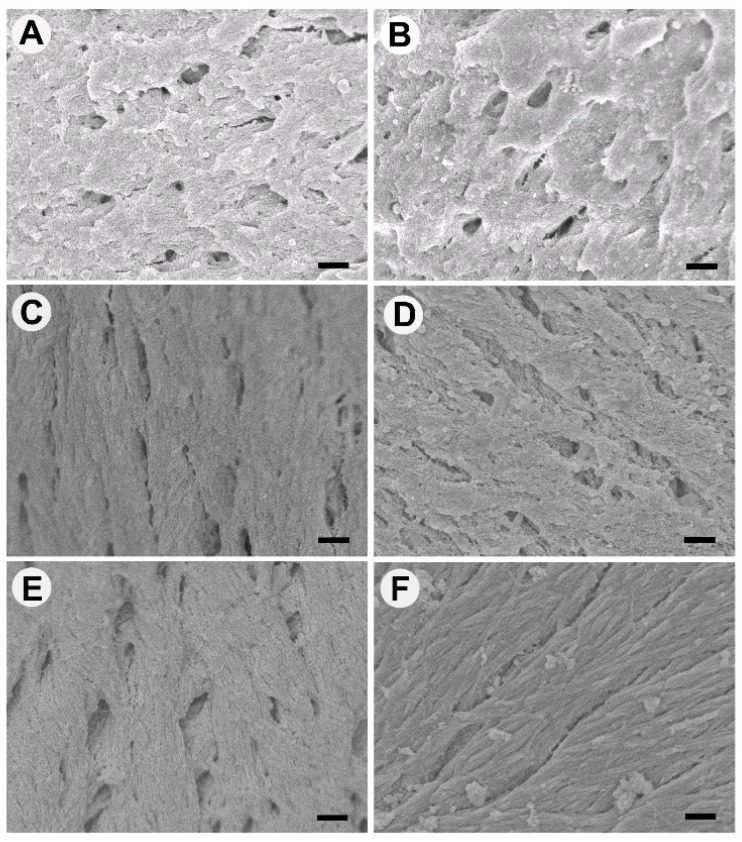
Scanning electron microscopy images of the surfaces of bone scaffolds heat-treated at (**A**) 100 °C, (**B**) 130 °C, (**C**) 160 °C, (**D**) 190 °C, (**E**) 220 °C, (**F**) Bio-Oss^®^. Scale bar = 1 µm. Representative images of N = 4.

The surfaces of the bone processed at different temperatures were imaged using SEM and are shown in Figure 1 (lower magnification images shown in Figure S1). Bone treated at 100 °C and 130 °C lacked the uniformity of surface patterns, with unevenly distributed isotropic appearance, shallow holes, and agglomerated ridges (Figure 1A,B). Bone treated at 160 °C had an anisotropic smoother surface, with distinct continuous lines, rather than overlaid ridges as observed at lower temperatures. The bone at 160 °C also had elliptical, regular holes (Figure 1C). Bone treated at 190 °C was similar to 160 °C; however, the surface appeared rougher with surface particulates; additionally, a fibrillar layer was visible beneath the top surface (Figure 1D). Bone treated at 220 °C had the appearance of an interwoven material with an upper surface of lines in one direction and the lower layer forming in the other direction (Figure 1E). Bio-Oss^®^ was significantly different in appearance (Figure 1F), with smooth fibrillar strands and micrometer-scale aggregates adsorbed onto the surface of the material. Images of the bone surface topography were also recorded at the cut surface sites, where osteoclasts did not adhere and are shown in Figure S2. The cut surfaces lacked the features described above found on natural surfaces and appeared similar to an agglomerated, condensed mass of bone aggregates.

### 3.2. Osteoclast Activity and Proliferation

**Figure 2:**
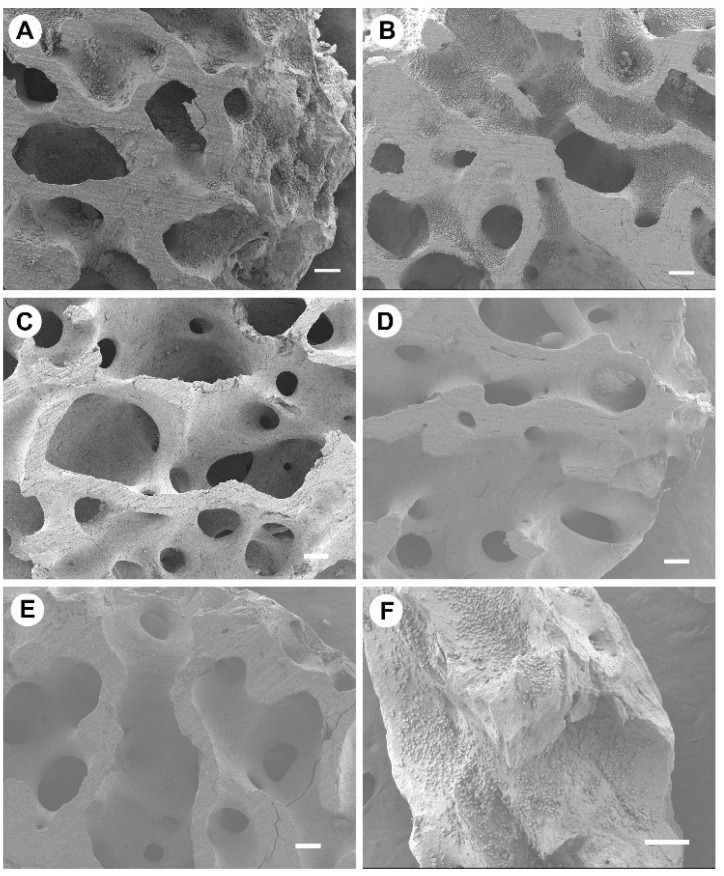
Scanning electron microscopy images of osteoclasts cultured on bone scaffolds heat-treated at (**A**) 100 °C, (**B**) 130 °C, (**C**) 160 °C, (**D**) 190 °C, (**E**) 220 °C, (**F**) Bio-Oss^®^. Scale bar = 200 µm. Representative images of N = 4.

The proliferation and morphology of osteoclasts growing on heated bone groups were observed via SEM imaging and are shown in Figure 2. High quantities of osteoclasts were present on 100 °C and 130 °C bone groups; cells grew as aggregated communities within bone trabecula rather than on the flat upper surface of bone discs. These cells can be distinguished as the darker grey aggregates, which are evident in Figure 2A,B,F, and a small quantity in Figure 2C; only the smooth bone surface is evident in Figure 2D,E. No cells, or <20, were found on 160 °C, 190 °C, and 220 °C treated bone specimens. Osteoclast growth on Bio-Oss^®^ was variable, and cells grew evenly across ~70% of the replicate granules; however, ~30% of granules exhibited no osteoclast presence. Attachment of cells was visually consistent across the replicate 100 °C and 130 °C bone specimens. Osteoclast morphology at 100 °C and 130 °C had a bimodal population of small, rounded cells and flattened elongated cells (Figure S3A–D). Osteoclasts that were adhered to Bio-Oss^®^ were typically rounded and small (Figure S3E,F). RAW 264.7 cells attached to and proliferating on the bone did not show pit or trench formation.

Osteoclast differentiation was confirmed on plastic well controls via the presence of TRAP-positive (stained pink) and multinucleated cells (Figure S4). TRAP is a cytochemical marker that is indicative of osteoclast function and degree of bone resorption. TRAP production (Figure S5) from cells on heated bone scaffolds was relatively low when compared to typically reported concentrations, and produced quantities were not significantly different between 100 °C, 130 °C, and 160 °C. TRAP activity was present in association with 190 °C and 220 °C thermal treated specimens, where cell presence was <20 cells (observed via SEM imaging). Bio-Oss^®^ exhibited higher concentrations of TRAP across the tested groups with increased variability between replicates.

### 3.3. Osteoblast DNA Quantity and Cell Count

**Figure 3:**
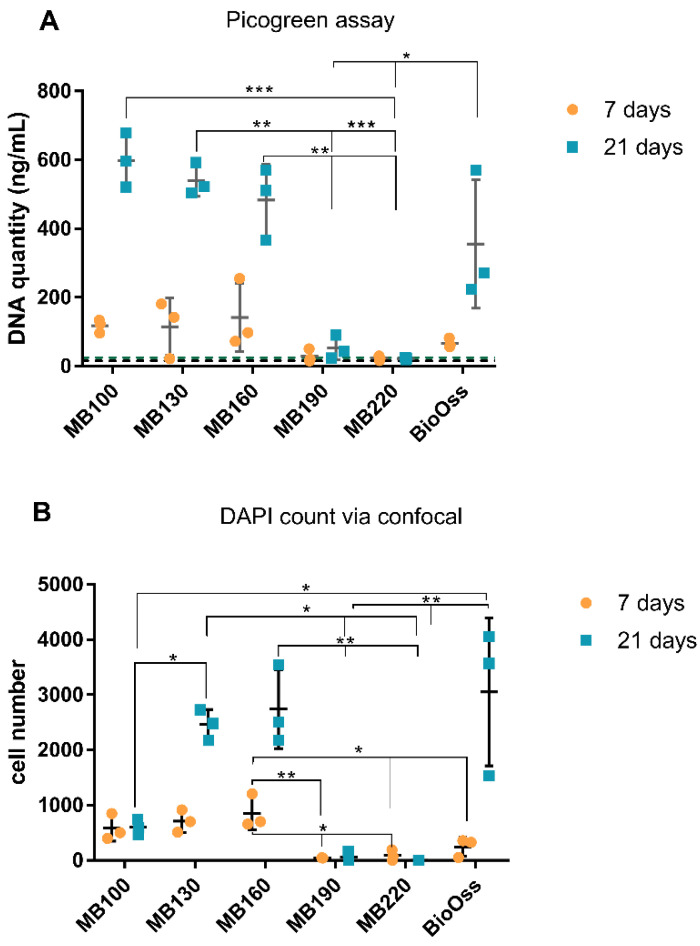
Osteoblast quantities at 7 and 21 days on bone scaffold specimens using picogreen assay (**A**), and cell counting of DAPI stained nuclei using confocal laser scanning microscopy and Fiji:image J analysis (**B**). N = 3. Results expressed as mean ± SD. Dotted lines: 7-day negative control (black) and 21-day negative control. Significant values * *p* ≤ 0.05, ** *p* ≤ 0.01, *** *p* ≤ 0.001.

**Figure 4:**
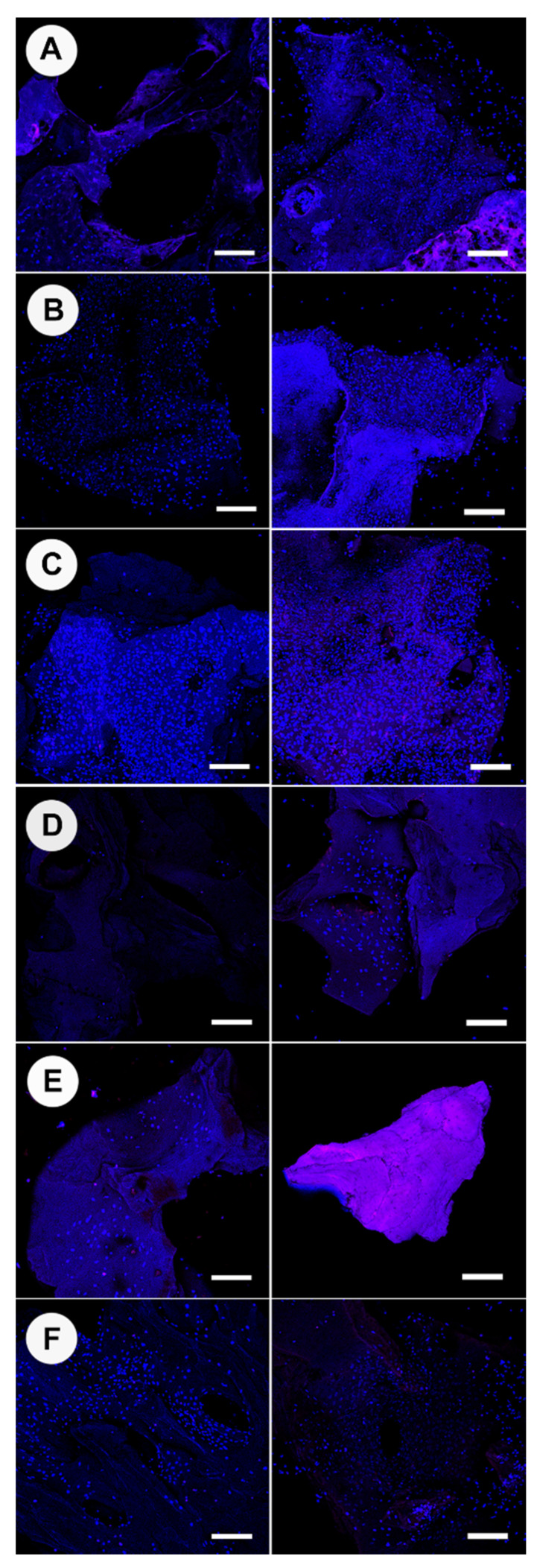
Confocal laser scanning microscopy (CLSM) of DAPI stained osteoblast nuclei (blue) at 7 (left) and 21 days(right) on bone scaffold specimens processed at 100 °C (**A**), 130 °C (**B**), 160 °C (**C**), 190 °C (**D**), 220 °C (**E**), Bio-Oss^®^ (**F**). Scale bar = 200 µm. Representative images of N = 3.

Osteoblast cell numbers as determined from DNA quantitation (Picogreen assay) and DAPI counts on heated bone scaffolds at 7 and 21 days are shown in Figure 3. Overall, the seven-day timepoint produced lower quantities of recoverable DNA than the 21-day culture (Figure 3A). Bone heat-treated at 100 °C, 130 °C, and 160 °C for 7 days had higher DNA quantities compared to all other heat treatment groups. Bone heat-treated at 190 °C and 220 °C exhibited no presence of DNA, with values consistent with media-only control wells. At 21 days, 100 °C, 130 °C, and 160 °C continued to demonstrate the highest DNA quantities amongst tested groups, along with Bio-Oss^®^. Bone heat-treated at 100 °C demonstrated the highest mean increase in DNA quantity between the 7-day and 21-day time points. The Bio-Oss^®^ specimens also demonstrated a noticeable increase in cell number between 7 and 21 days but had high variability between replicates. Proliferation was also measured via cell counting using DAPI with confocal imaging and subsequent image analysis using Fiji:Image J (Figure 3B and Figure 4). Bone heat-treated at 130 °C, 160 °C, and Bio-Oss^®^ demonstrated the highest number of cells at 21 days. Bio-Oss^®^ exhibited the greatest variability amongst samples, which was consistent throughout experiments. When observing overall cell growth trends between the two analysis methods, picogreen assay and cell counting, both methods reported similar cell quantities for all groups except for bone treated at 100 °C, which did not report an increase in cell number at 21 days when derived from confocal analysis.

**Figure 5:**
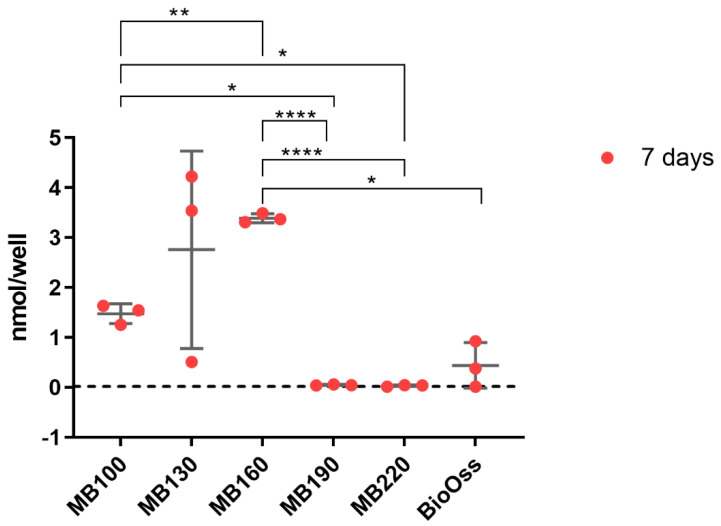
Alkaline phosphatase production determined from osteoblasts cultured on bone scaffold specimens at 7 days. N=3. Results expressed as mean ± SD. Dotted line: 7−day negative control. Sig−ificant values * *p* ≤ 0.05, ** *p* ≤ 0.01, **** *p* ≤ 0.0001.

ALP production levels from osteoblasts on heated bone specimens at 7 days are shown in Figure 5. ALP production was significantly increased when the bone was heated treated at 160 °C compared to 100 °C (*p* = 0.0064). Cells cultured on bone heated at 130 °C also demonstrated higher mean quantities of ALP but greater viability between samples. No ALP production was recorded from bone heat-treated at 190 °C and 220 °C, which was consistent with the lack of osteoblast growth observed on these bone specimens. ALP concentrations recorded from cells on bone heat-treated at 100 °C were significantly higher than bone heat-treated at 190 °C and 220 °C but were not significantly different from Bio-Oss^®^.

**Figure 6:**
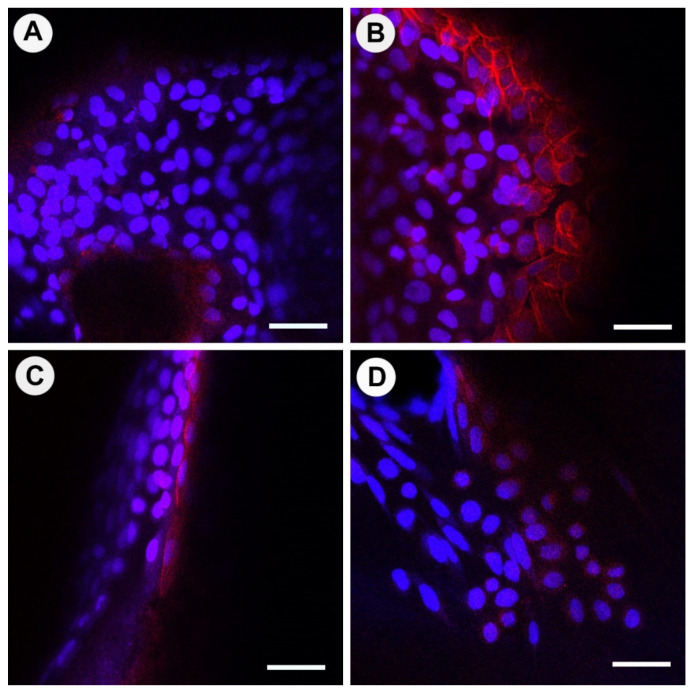
Confocal laser scanning microscopy (CLSM) of osteoblast actin filaments (red) and osteoblast nuclei (blue) cultured in osteogenic media for 21 days on bone scaffolds heat-treated at 100 °C (**A**), 130 °C (**B**), 160 °C (**C**), and Bio-Oss^®^ (**D**). Scale bar = 50 µm.

Images of osteoblasts with Alexa Fluor™ 647 Phalloidin staining of actin filaments are shown in Figure 6 (osteogenic media) and Figure S6 (non-osteogenic media). Cells attached to bone heat-treated at 100 °C appeared to have large nuclei and trapezoid skeletal actin structures; these structures were non-distinct and net-like (Figure 6A). Cells attached to bone heat-treated at 130 °C had actin skeletal structures that formed a connected sheet network with large trapezoid and circular cells; cells were also clearly undergoing mitosis (Figure 6B). Cells attached to bone heat-treated at 160 °C were elongated and organized into continuous strips (Figure 6C). Bio-Oss^®^ osteoblast cells were bimodal in actin skeletal appearance, approximately half were rounded, and half were spindle-like with two processes at either end of the nuclei (Figure 6D).

**Figure 7:**
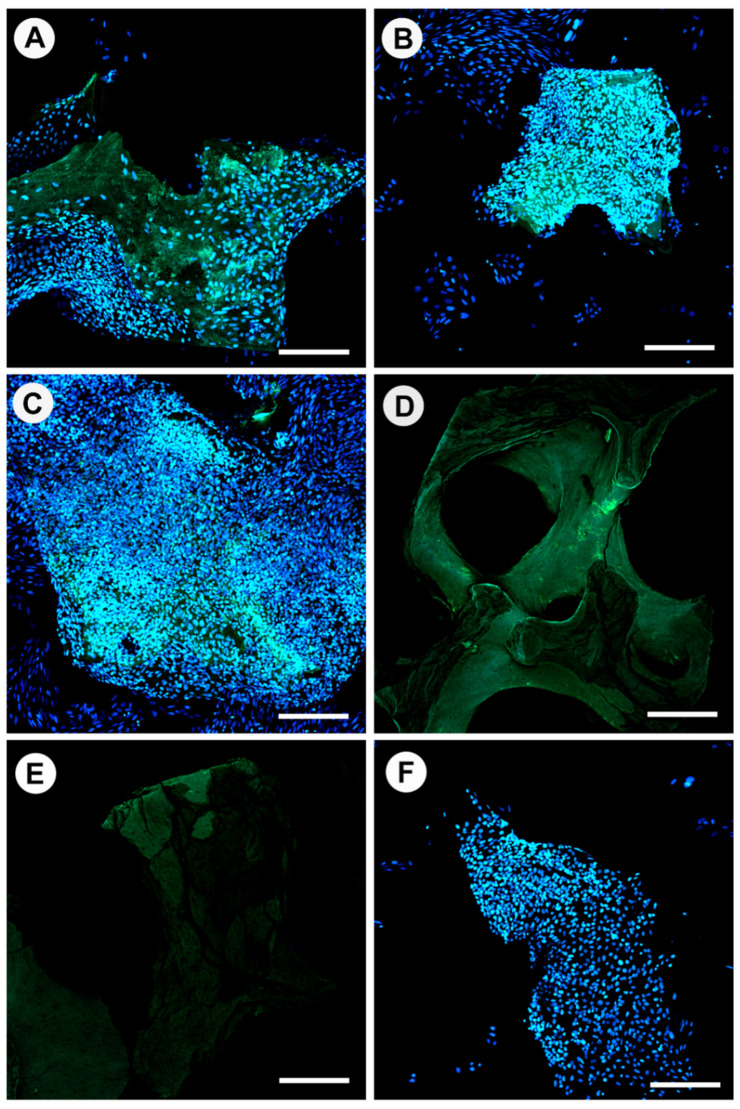
Confocal laser scanning microscopy (CLSM) of osteoblast nuclei (blue) and osteocalcin (green) cultured in osteogenic media for 21 days on bone scaffolds heat-treated at 100 °C (**A**), 130 °C (**B**), 160 °C (**C**), 190 °C (**D**), 220 °C (**E**), Bio-Oss^®^ (**F**). Scale bar = 200 µm.

Osteocalcin, a small non-collagenous protein produced exclusively by osteoblasts, is generally regarded as a marker of bone formation. The presence of osteocalcin on the heat-treated bone specimens is shown in Figure 7 and can be distinguished as green fluorescence. OC production appeared limited and non-matured for all specimens investigated, and when present, OC was found to be beneath cells (DAPI stained nuclei). OC presence was more distinct in areas where cells were particularly dense and overlapped. Cells attached to bone heat-treated at 130 °C and 160 °C had proliferated further than the margins of the bone granules and had connected the granule to the bottom of the well; every nucleus observed was associated with an area of OC production. Bone heat-treated at 190 °C had residual OC evident in small quantities on the bone; however, cells did not attach. Bone heat-treated at 220 °C showed no presence of OC. Bio-Oss^®^ possessed the least OC associated with cells amongst the specimens. It was interesting to observe that some groups of bone granules had adhered to the culture well due to cell sheeting from granule to the well surface; in particular, 100% of granules for 160 °C were immobile, 60% for 130 °C, 40% for 100 °C, and full mobility was seen for 190 °C, 220 °C, and Bio-Oss^®^ within wells.

The gene expression levels as represented by Cq values are shown in Figure S7, where a higher expression is a lower Cq. No gene was undetected (mean Cq = 40). In general, the osteogenic and non-osteogenic media and 7- and 21-day time periods showed little difference in resulting Cq values. *BGLAP* had higher Cq values and, therefore, lower cDNA quantities compared to the other genes of interest. *ALPL* and *COL1A1* demonstrated the lowest Cq values and thus high cDNA quantities across the two time periods.

**Figure 8:**
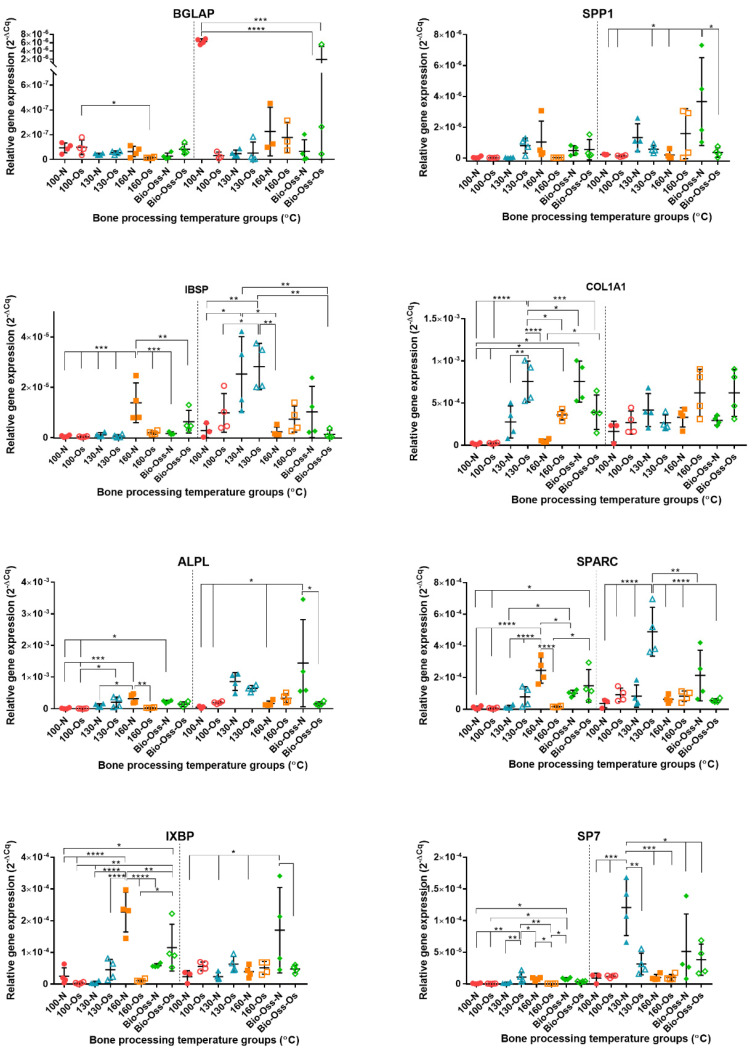
Relative qRT2−PCR gene expression of genes of interest in osteoblasts cultured on bone processed at different temperatures. The dashed line indicates 7−day expression on the left of the axis and 21−day expression on the right of the axis. N = 4. Results expressed as mean ± SD. Significant values * *p* ≤ 0.05, ** *p* ≤ 0.01, *** *p* ≤ 0.001, **** *p* ≤ 0.0001.

The relative gene expression between test groups for each gene of interest is presented in Figure 8. Typically, an increase in gene expression was observed at 21 days compared with 7 days. As with Cq values, there was no clear trend of gene expression with the use of either non-osteogenic or osteogenic media. The lowest relative gene expression levels were found in the lowest temperature group, 100 °C, except for *BGLAP* in non-osteogenic media, where there was a highly significant increased expression. Bio-Oss^®^ tended to show large variation across all genes of interest between replicates. *COL1A1* transcript was high in all groups at 21 days, which reflects the low Cq values determined initially. At 7 days, the most “active” test group in terms of significantly higher GOI expression was evident for 160 °C non-osteogenic media (*BGLAP*, *IBSP, ALPL*, *SPARC*, *OXBP*), and 130 °C bone in osteogenic media for *COL1A1* and *SP7*. At 21 days, there was significantly increased expression for 130 non-osteogenic and osteogenic, 160 osteogenic, and Bio-Oss^®^ non-osteogenic, which were equivalent for *IBSP*, *SPP1*, *ALPL*, and *IXBP*; however, 130 osteogenic showed significantly higher expression of *SPARC* and *SP7*.

## 4. Discussion

Small scale changes in thermal processing temperatures of 100 °C, 130 °C, 160 °C, 190 °C, and 220 °C were performed on bovine bone, and the resultant effects on osteogenesis were assessed. Osteoclast and osteoblast activity on the bone surfaces were compared to a clinical control Bio-Oss^®^.

The critical interplay between bone resorption and bone formation and the respective coordination of osteoclasts and osteoblasts is required for successful bone remodeling. If an incorrect balance between the two occurs, it can result in reduced bone strength. With respect to bone grafting materials, unbalanced processes can affect osseointegration, producing either non-resorbed graft material, graft material that resorbs too quickly, or material failure. In a previous investigation, the use of an in vivo ovine model demonstrated that Bio-Oss^®^ underwent minimum to no resorption, forming “islands” of isolated grafting material as seen with bone apposition, whereas a commercial MoaBone^®^ product (M-Sphere^®^) was rapidly resorbed and replaced with connective tissue [27]. It is known that the rate and extent of material incorporation are dependent on the type of graft material, producing a varying level of healing and mechanical stability [1,2,3,4]. In particular, it was suggested that substitute bone grafts are limited to osteoconductive capabilities, with osteoinductive properties being a sought-after attribute often found with more biomimetic grafts. At present, despite good clinical outcomes, typically, the center of graft materials remains unremodelled, and there is a lack of sufficient integration [32]. In light of these drawbacks, bone grafts are continually optimized to produce materials that are osteoinductive, osteoconductive, and undergo osteogenesis in order to provide the best clinical outcomes. When optimizing osteogenic materials, it is important to observe how material changes can facilitate interactions with bone-forming cells, and despite numerous new and existing substitute grafts, along with in vitro and in vivo trials, there is limited knowledge of the material characteristics and predicted cell response [32].

Osteoclast attachment was prevalent on bone graft material processed at lower temperatures of 100 °C and 130 °C; however, there was little evidence of resorptive cell behavior at 7 days. At ≥160 °C, osteoclasts had very limited or no attachment. The attachment behavior of bone cells was associated with the surface morphology of graft materials [32]. When correlating the osteoclast presence to bone surface structure, osteoclasts appeared to prefer the rough, irregular surfaces produced at lower temperatures. Comparable osteoclasts cell quantities were observed for 160 °C and Bio-Oss^®^, where both graft surfaces appeared most morphologically similar using SEM. The osteoclasts on Bio-Oss^®^ were rounded and singular, suggesting a lack of resorptive activity compared to the larger, flat, and ruffled cells observed on 100 °C and 130 °C New Zealand bone constructs. Bio-Oss^®^ with seeded osteoclasts (RAW 264.7 cells) were shown to have singular rounded osteoclasts in the literature previously and is partnered with slow resorption but high dimensional maintenance [24,26]. When observing the variability of Bio-Oss^®^, increased osteoclast attachment was seen in areas prone to increased surface aggregates rather than smooth areas. It was found that the sealing zones of osteoclasts preferentially develop around surface protrusions [33], which was consistent with our findings.

Osteoclasts and osteoblasts did not attach to cut surfaces and congregated on the natural surfaces of the bone. We believe this demonstrates the importance of surface morphology as the cut surface was compacted and appeared to deter cell attachment—this would suggest processing methods that ensure maximum natural surface area would be most beneficial for in vitro and in vivo cell attachment. Osteoblast activity was most prevalent on 130 °C and 160 °C heat-treated bone. The processed bone at temperatures ≥190 °C showed no osteoblast attachment and limited attachment at 100 °C—highlighting a possible “Goldie locks” region. When assessing osteoblasts proliferation, attachment, cell sheeting, and osteocalcin production were all more evident for 130 °C and 160 °C treated bone. In terms of osteogenic gene activity, again, 130 °C and 160 °C showed significantly increased activity when compared to the other processing temperatures. Similar activity was observed for Bio-Oss^®^ at 21 days but not at the earlier 7-day time point; additionally, Bio-Oss^®^ had the highest variability in all assay results. It is considered that this variability may be due to the range Bio-Oss^®^ of particles within one given lot resembling both cortical and cancellous bone, which was reported by Dumitrescu et al. [25]. Microroughness of graft surfaces is viewed as superior to smooth surfaces in terms of osteoblast-induced bone integration [32,34]; however, changes in the nano or micro surface properties can influence cell targeting behavior [32]. Anselme and Bigerelle stated that human osteoblasts are more sensitive to the organization and morphology of the roughness rather than to its amplitude [35] in reference to titanium implants. As suggested by Anselme and Bigerelle and Rabel et al., our findings are in agreement that osteoblasts are preferentially bound and differentiated on materials with anisotropic, regular alignment providing contact guidance for osteoblasts [32,35] and were reduced on isotropic surfaces [32].

In general, the previous literature reports of temperature changes resulting in altered cellular activity were performed at high calcination temperatures such as 700–1200 °C [12,36,37]. Comparative to our findings, reports find that higher temperatures (1200 °C) can result in higher cell proliferation and higher protein levels of bone sialoprotein, osteocalcin, and osteonectin [38,39]. However, osteonectin and type I collagen mRNA expression were not significantly altered by heating temperature [38]. Laquerriere et al. (2001) reported higher surface toxicity associated with cells at lower bone treatment temperatures of 600 °C [40]. EU guidelines of animal tissue derivatives used in medical devices guidelines at an acceptable minimum of 800 °C reduce the risk of the transmission of Transmissible Spongiform Encephalopathies (TSEs) (ISO ISO 22442-1:2020) [41] such as prions. Bio-Oss^®^ is heated at 350 °C [22,37] and is typically referred to as a low-temperature graft [21] compared to similar substitute grafts produced at higher sintering temperatures such as PepGen P-15^®^ (1100 °C), Endobon^®^ and Cerabone^®^ (>1200 °C), and Algipore^®^ (700 °C) [1]. Higher temperature processing produces higher crystalline graft materials, which usually demonstrate slow resorption and low mechanical strength; in comparison, the relatively low production temperature of Bio-Oss^®^ would suggest a fast resorption period [36]; however, this was not observed in vivo with regards to Smith et al. [27]. Morroni et al. investigated an anorganic bovine bone graft material that was prepared using thermal processing at 350 °C, which is similar to Bio-Oss^®^ and is consistent with other commercial products. They demonstrated slow resorption in vivo (particles retained after 90 days) and recognized heated xenografts were clinically found to persist several years after implantation [42], which could be due to the limited osteoclast interaction in vivo. Similarly, we found osteoclasts on Bio-Oss^®^ to be rounded and small, rather than the flat ruffled morphology attributed to active resorption. Furthermore, Jensen et al. found that 32 ± 9.6% of Bio-Oss^®^ particles remained intact 14–80 months after placement of the graft material, and 70.3 ± 14.5% of the particle surfaces were covered with bone [43], reinforcing that the center of the bone grafts is not remodeled. Using a prion-free bovine source may enable the possibility to use the low temperatures associated with resorptive osteoclast behavior and preservation of the bone structure. In addition, it is suggested that using a lower temperature would be beneficial for the preservation of the original collagen fibril structures, supporting further neosynthesis and providing templates for subsequent mineralization. Ghanaati et al. found that organic remnants did not induce inflammatory results in cells that could encourage the use of collagen preservation. They also suggested that a high-temperature heating process reduces the material porosity and melts the lamellar structure, impairing material wettability and osteogenic cell attachment [21].

A lack of sufficient interaction with bone cells in vitro could suggest suboptimal properties and thus failure to permit integration and production of new bone to support and replace the grafting material. When observing the osteogenic or osteoconductive nature of potential bone scaffolds, it is beneficial to investigate gene expression involved in the stepwise processes of osteogenic differentiation. Alkaline phosphatase, osteocalcin, osteonectin, bone sialoprotein, osteopontin, osteonectin, osterix, and type I collagen are important proteins expressed during osteogenesis and the production of osseous matrix and calcification. Type I collagen (*COL1A1*) is the major bone matrix protein constituting 90% of the organic matrix and can provide an anchorage structure for subsequent osseous proteins [44]. The early involvement of *COL1A1* was observed, and significant variation in expression at the 7-day time point was evident; there were, however, no differences seen at 21 days. *COL1A1* expression was a positive indicator for the bone treatment groups that demonstrated significant osteogenic gene expression at 21 days. Alkaline phosphatase is produced early in osteoblast differentiation; ALP plays an important role in hydrolyzing inorganic pyrophosphate to inorganic phosphate, blocking the inhibition of hydroxyapatite formation, and supplying Pi for hydroxyapatite production [45]. As with *COL1A1*, significant regulation of *ALPL* gene expression was more evident between groups at 7 days. Gene expression levels of *ALPL* were consistent with the secreted alkaline phosphatase levels. Bone sialoprotein (*IBSP*) is associated with bone growth at the early stage through the nucleation of hydroxyapatite [46]; however, higher overall expression was prevalent at 21 days. Osteonectin (*SPARC*) participates in mineralization but also regulates extracellular matrix mineralization [47]. Osteopontin (*SPP1*) allows regulation of osteoclastic activity, activation of osteoprotegerin expression, and OPN-mediated bone remodeling [48].

Osterix (*SP7*) is considered an essential transcription factor regulating osteoblast differentiation and bone formation; when absent, no cortical bone and no bone trabeculae are formed. Interestingly osterix expression alone is sufficient to activate the osteocalcin gene and *COL1A1,* suggesting its very early involvement in the cell differentiation processes [49]. Higher osterix expression was evident for bone processed at 160 °C and Bio-Oss^®^ in non-osteogenic media, and 130 °C in osteogenic media at the earlier 7-day time point. There appeared to be a later 21-day expression for the 130 °C non-osteogenic media group. Bone treatment groups with significantly increased early expression of *IXBP* were consistent with those with increased osterix expression. The target transcription factor X-box binding protein 1 (*XBP1*) is essential for bone morphogenic protein 2-induced osteoblast differentiation through the promotion of osterix transcription [50]. Osteocalcin (*BGLAP*) was reported to drive the necessary alignment of biological apatite crystallites parallel to collagen fibers [51] and regulation of the rate of mineral maturation [52]; it is considered a late osteoblast marker. Cellular proliferation is typically down-regulated upon differentiation, and gene expression associated with extracellular matrix maturation is induced, with subsequent markers of mineralization. Osteocalcin was significantly upregulated at the 21-day time points for 100 °C non-osteogenic only, which had not shown a significantly higher expression level for the other investigated genes of interest. In addition, the 100 °C processed bone had lower cell proliferation levels when compared to bone treated at 130 °C and 160 °C. It is possible that the surface of the 100 °C treated bone was conductive of osteocalcin gene expression only, as the surface may have been obstructed by adsorbed osteogenic media elements producing significant expression differences between media types. In particular it was reported that unprocessed hydroxyapatite bone has high surface free calcium, and therefore adsorbs higher protein quantities, such as albumin, thus the degree of free calcium ion ligands are reduced with increased heat processing temperatures [39]. The ability of the surface to adsorb proteins and glycosaminoglycans was correlated with higher matrix protein production from seeded osteoblast cells, although high sintering temperatures were seen to induce faster rates of cell differentiation and mineralization [39].

## 5. Conclusions

Of all heat treatments investigated, 130° C and 160 °C possessed the highest osteoblast proliferation, ALP activity, and osteogenic gene expression. At these temperatures, osteoblasts appeared larger, stretched, and formed sheets, facilitated by the anisotropic characteristic of the surface; these phenotypes are typically consistent with promising bone formation. Osteoclasts demonstrated a preference for roughened, protruding surfaces which were correlated with lower bone processing temperatures. Both observed cell characteristics would suggest that the formation of new bone around the processed bone scaffolds would be highest for these two highlighted temperatures of 130 °C and 160 °C. A lower variability of cellular interaction and biological function was evident for the New Zealand bone materials compared to the commercial control, which is beneficial for predicted bone regeneration in vivo. In conclusion, we determined that incremental changes to the method of bone production, in this case, thermal processing, can have significant impacts on the osteogenic differentiation and behavior of attachment of osteoblasts and osteoclasts in vitro and demonstrated how relatively small changes in temperature result in marked alteration in the materials attributes in a graft application. Such findings could influence the production methods of regenerative biomaterials with the goal of improving clinical outcomes in vivo. A further in vivo calvarial rabbit model was performed using the highlighted bone graft materials described in this manuscript in order to understand the histomorphometric responses of tissues, new bone growth, and monitoring of graft presence, addressing the limitations of in vitro monocultures.

## 6. Patents

All authors are named inventors on a provisional patent application “Bone graft material” New Zealand 781191.

## Figures and Tables

**Table 1:** Pressure recorded at each thermal bone-processing temperature within the stainless-steel vessel.

Temperature (°C)	Pressure (Bar)
100	1.01
130	2.69
160	6.22
190	12.93
220	24.58

## Data Availability

The raw/processed data required to reproduce these findings cannot be shared at this time as the data also form part of an ongoing study. Data may be requested from authors.

## Supplementary Materials

The following supporting information can be downloaded at: https://www.mdpi.com/article/10.3390/ma15072504/s1, Figure S1. Scanning electron microscopy images of the surfaces of bone scaffolds heat-treated at (A) 100 °C, (B) 130 °C, (C) 160 °C, (D) 190 °C, (E) 220 °C, (F) Bio-Oss^®^. Scale bar = 10 µm. Representative images of N = 4. Figure S2. Scanning electron microscopy images of the cut surfaces of bone scaffolds heat-treated at (A) 100 °C, (B) 130 °C, (C) 160 °C, (D) 190 °C, (E) 220 °C, (F) Bio-Oss^®^. Scale bar = 1 µm. Representative images of N = 4. Figure S3. High magnification scanning electron microscopy images of osteoclasts cultured on bone scaffold specimens heat-treated at 100 °C (A,B), 130 °C (C,D), and BioOss (E,F). Scale bar =100 µm (left) and 10 µm (right). N = 4. Figure S4. Light microscopy images showing tartrate-resistant acid phosphatase positive and multinucleated osteoclasts (pink) on plastic wells at (A) high magnification and (B,C) duplicate wells. Figure S5. Tartrate-resistant acid phosphatase activity determined from the supernatant of osteoclasts cultured on bone scaffold specimens at 7 days. N = 4. Results expressed as mean ± SD. Dotted line represents the negative control value. Figure S6. Confocal laser scanning microscopy (CLSM) of osteoblast actin filaments (red) and osteoblast nuclei (blue) cultured in non-osteogenic media for 21 days on bone scaffolds heat-treated at (A) 100 °C, (B) 130 °C, (C) 160 °C, and (D) Bio-Oss^®^. Scale bar = 50 µm. Figure S7. Cq values of genes of interest expressed in osteoblasts (Saos-2 cells) cultured on bone processed at different temperatures at 7 and 21 days, in non-osteogenic (-N) and osteogenic media (Os). N= 4. Results expressed as mean ± SD. Dotted line at Cq 40 = not detected, dotted line at Cq 20 = highly expressed.
