# Supplementary material for: The Effect of Low-Temperature Thermal Processing on Bovine Hydroxyapatite Bone Substitutes, toward Bone Cell Interaction and Differentiation
Source: Materials (Basel). 2022 Mar 29;15(7):2504. doi: 10.3390/ma15072504 (PMC8999525; doi:10.3390/ma15072504)
Supplement: Supplementary file 1 [file materials-15-02504-s001.zip › materials-1633262-supplementary.pdf]

Supplementary material

The Effect of Low-Temperature Thermal Processing on Bovine Hydroxyapatite Bone Substitutes, toward Bone Cell Interaction and Differentiation

Gemma Claire Porter *, Dina Abdelmoneim, Kai Chun Li, Warwick John Duncan and Dawn Elizabeth Coates

Sir John Walsh Research Institute, Faculty of Dentistry, University of Otago, Dunedin 9016, New Zealand; abddi084@student.otago.ac.nz (D.A.); kc.li@otago.ac.nz (K.C.L.); warwick.duncan@otago.ac.nz (W.J.D.); dawn.coates@otago.ac.nz (D.E.C.)

* Correspondence: gemmacotton22@gmail.com

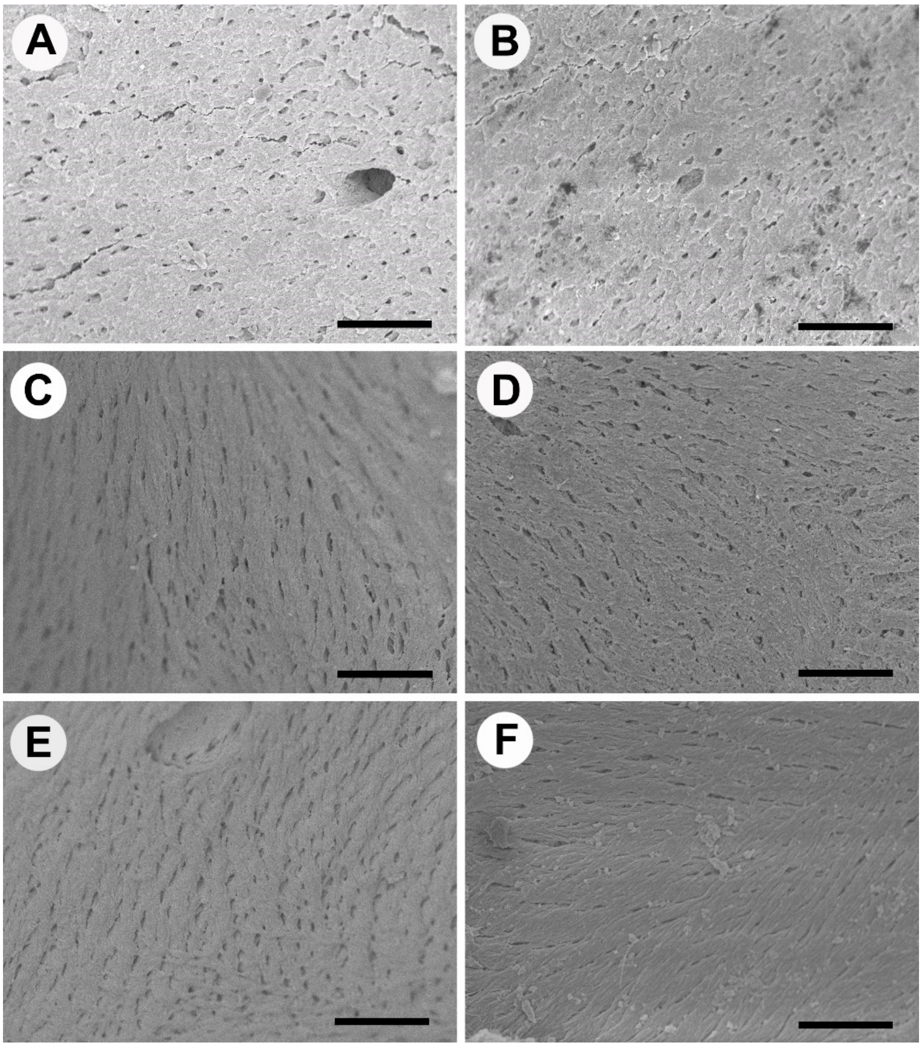

Figure S1. Scanning electron microscopy images of the surfaces of bone scaffolds heat-treated at (**A**) 100 °C, (**B**) 130 °C, (**C**) 160 °C, (**D**) 190 °C, (**E**) 220 °C, (**F**) Bio-Oss®. Scale bar = 10 µm. Representative images of N=4.

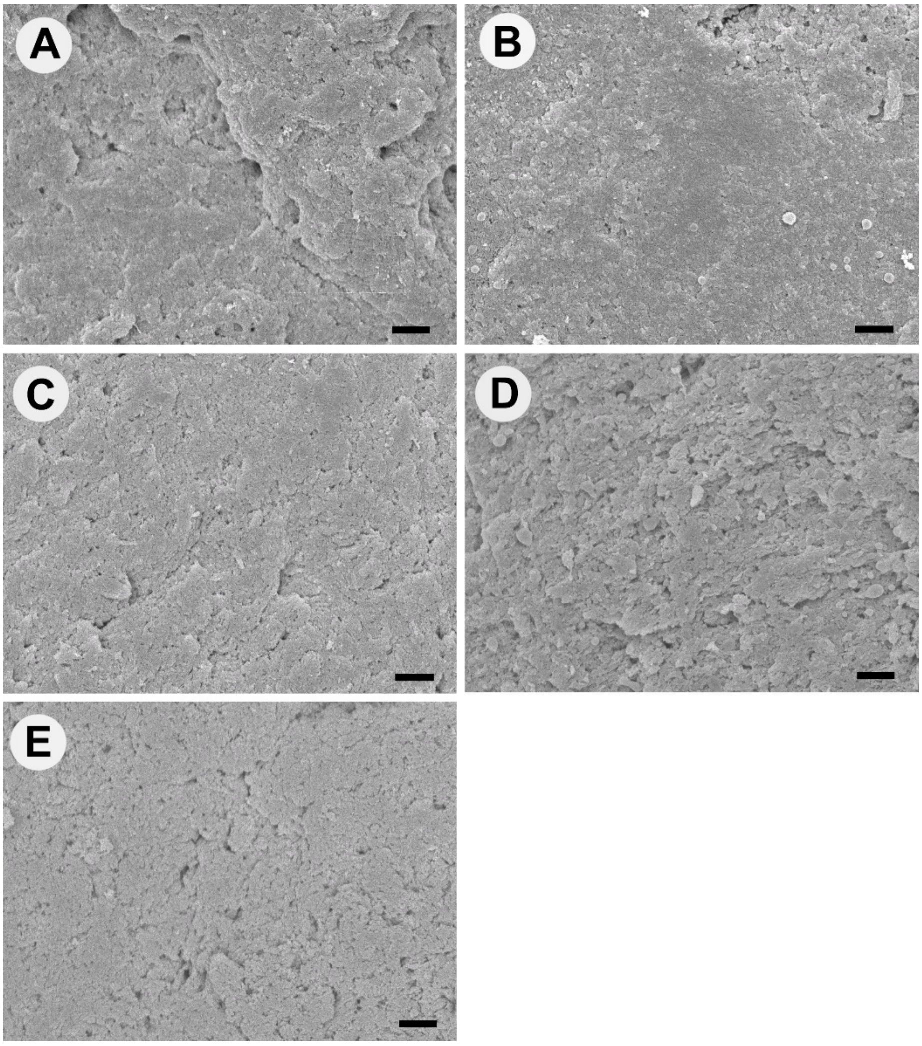

Figure S2. Scanning electron microscopy images of the cut surfaces of bone scaffolds heat-treated at (**A**) 100 °C, (**B**) 130 °C, (**C**) 160 °C, (**D**) 190 °C, (**E**) 220 °C. Scale bar = 1 µm. Representative images of N = 4.

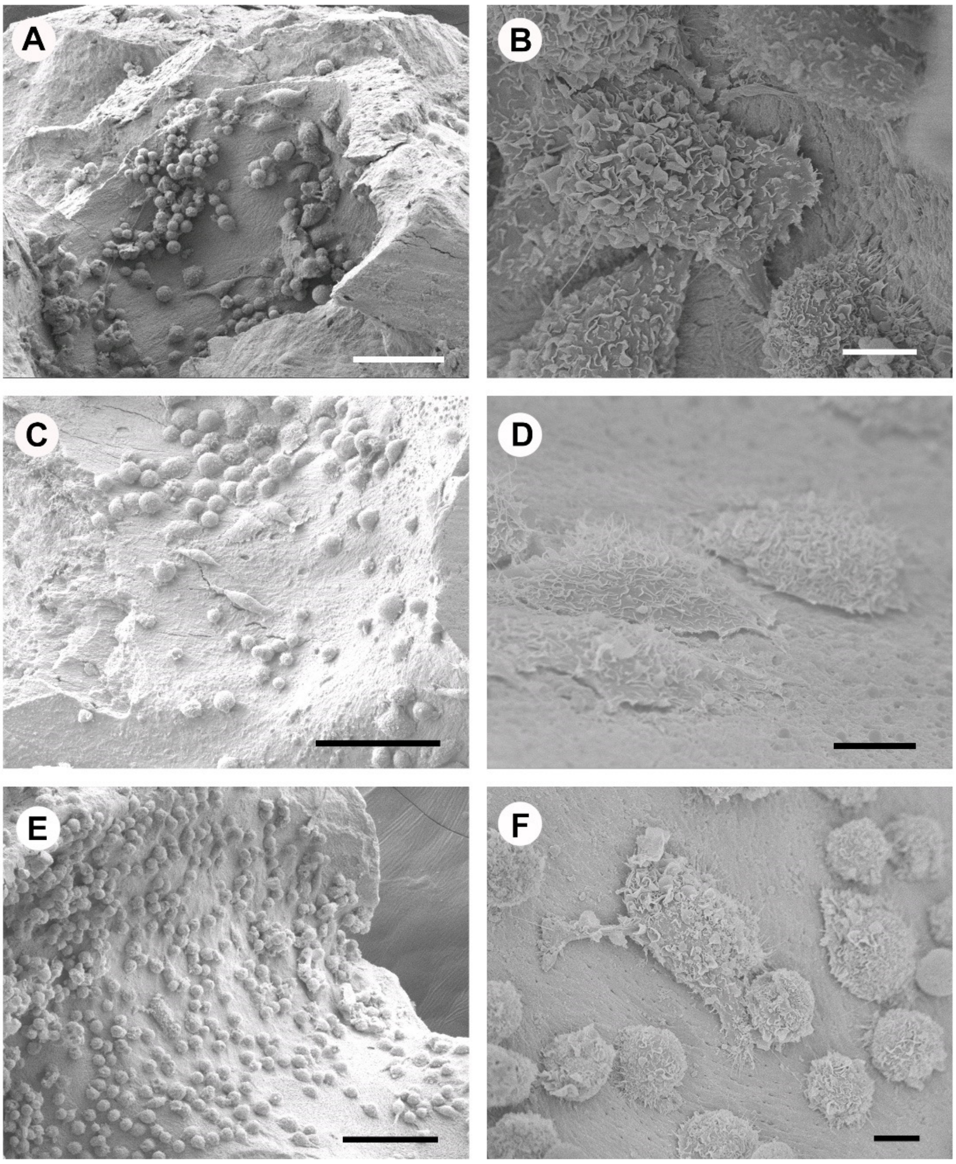

Figure S3. High magnification scanning electron microscopy images of osteoclasts cultured on bone scaffold specimens heat-treated at 100 °C (**A**,**B**) 130 °C (**C**,**D**) and Bio-Oss® (**E**,**F**). Scale bar =100 µm (left) and 10 µm (right). N = 4.

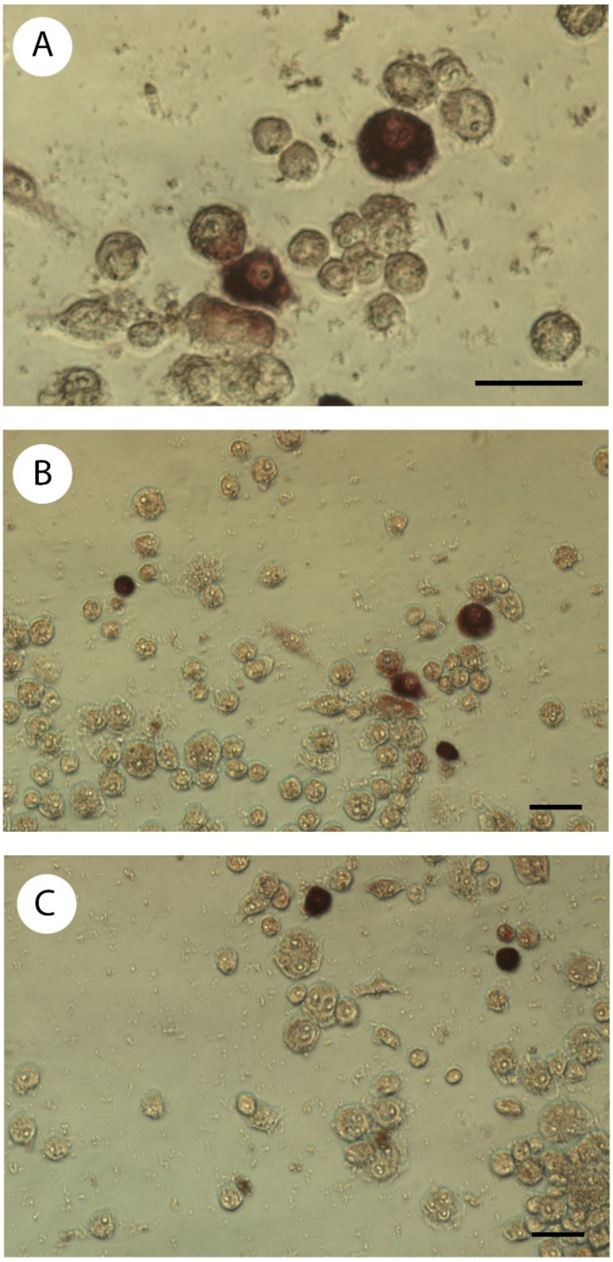

Figure S4. Light microscopy images showing tartrate-resistant acid phosphatase positive and multinucleated osteoclasts (pink) on plastic wells at (**A**) high magnification and (**B**,**C**) duplicate wells. Scale bar = 50 µm.

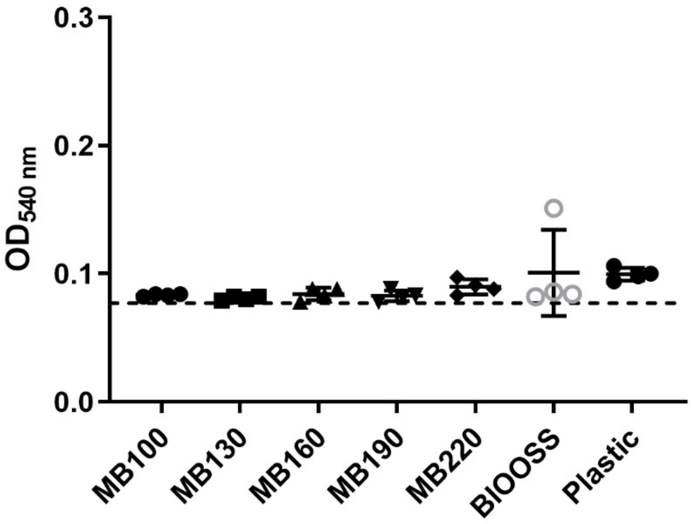

Figure S5. Tartrate-resistant acid phosphatase activity determined from the supernatant of osteoclasts cultured on bone scaffold specimens at 7 days. N = 4. Results expressed as mean ± SD. Dotted line represents the negative control value.

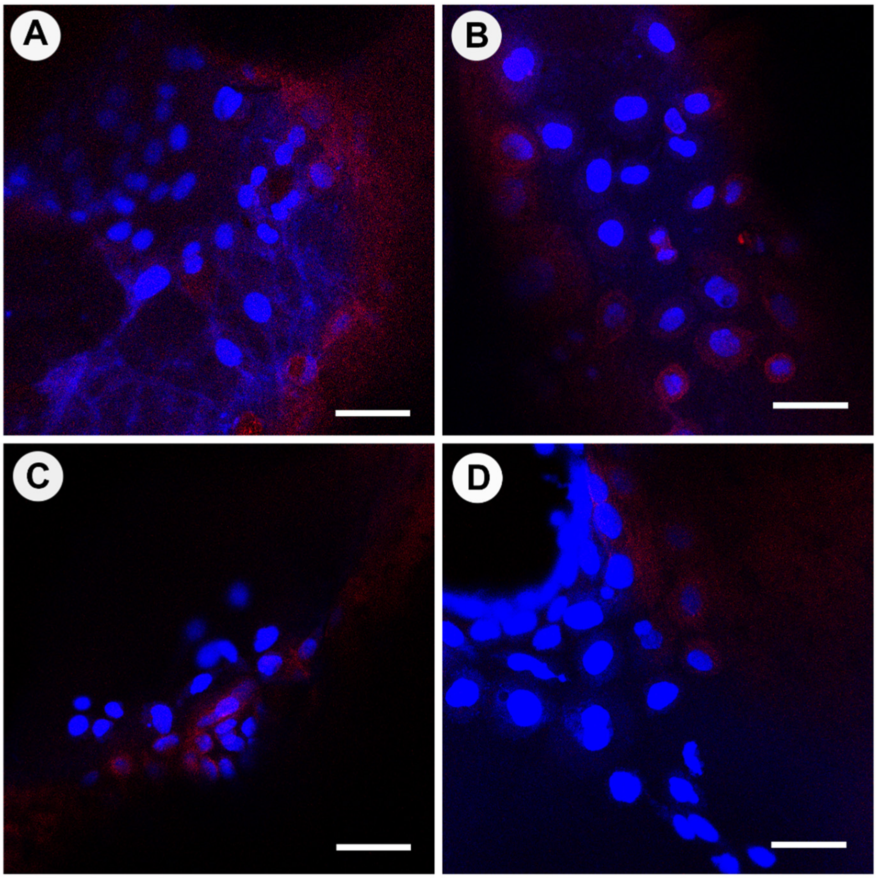

Figure S6. Confocal laser scanning microscopy (CLSM) of osteoblast actin filaments (red) and osteoblast nuclei (blue) cultured in non-osteogenic media for 21 days on bone scaffolds heat-treated at (**A**) 100 °C, (**B**) 130 °C, (**C**) 160 °C, and (**D**) BioOss. Scale bar = 50 µm.

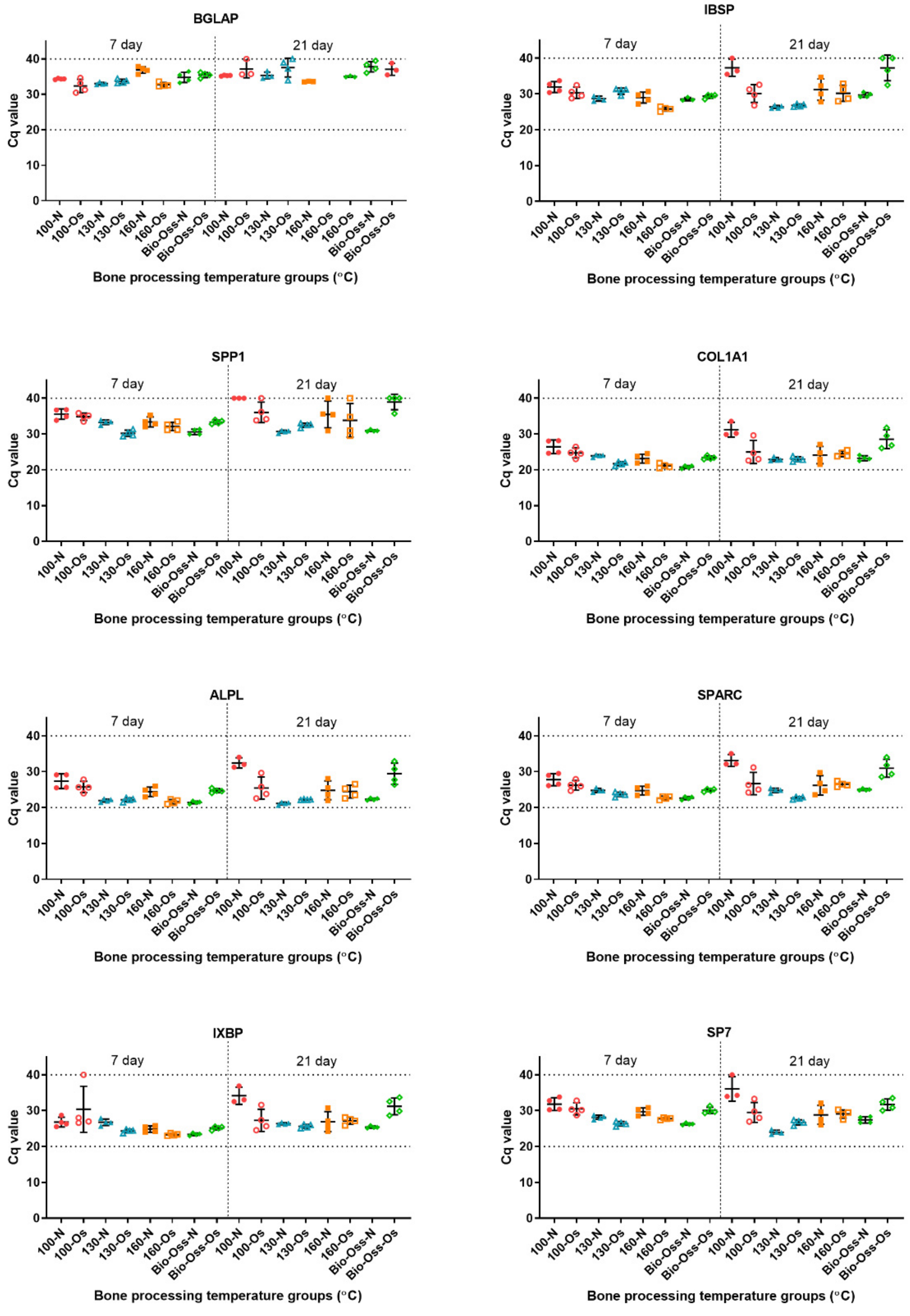

Figure S7. Cq values of genes of interest expressed in osteoblasts (Saos-2 cells) cultured on bone processed at different temperatures at 7 and 21 days, in non-osteogenic (-N) and osteogenic media (Os). N= 4. Results expressed as mean ± SD. Dotted line at Cq 40 = not detected, dotted line at Cq 20 = highly expressed.
